# Natural Killer T Cell-Targeted Immunotherapy Mediating Long-term Memory Responses and Strong Antitumor Activity

**DOI:** 10.3389/fimmu.2017.01206

**Published:** 2017-09-25

**Authors:** Nyambayar Dashtsoodol, Tomokuni Shigeura, Takuya Tashiro, Minako Aihara, Toshihiro Chikanishi, Hiromi Okada, Keigo Hanada, Hirokazu Sano, Akihiko Kurogi, Masaru Taniguchi

**Affiliations:** ^1^Laboratory for Immune Regulation, RIKEN Center for Integrative Medical Sciences, Yokohama, Japan; ^2^Core Research Laboratory, Mongolian National University of Medical Sciences, Ulaanbaatar, Mongolia; ^3^Faculty of Pharmaceutical Sciences, Niigata University of Pharmacy and Applied Life Sciences, Niigata, Japan

**Keywords:** natural killer T cell, tumor immunology, CD1d, immunotherapy, neoglycolipid, adjuvant activity, long-term memory responses

## Abstract

Current tumor therapies, including immunotherapies, focus on passive eradication or at least reduction of the tumor mass. However, cancer patients quite often suffer from tumor relapse or metastasis after such treatments. To overcome these problems, we have developed a natural killer T (NKT) cell-targeted immunotherapy focusing on active engagement of the patient’s immune system, but not directly targeting the tumor cells themselves. NKT cells express an invariant antigen receptor α chain encoded by *Trav11* (Vα14)-*Traj18* (Jα18) gene segments in mice and *TRAV10* (Vα24)-*TRAJ18* (Jα18) in humans and recognize glycolipid ligand in conjunction with a monomorphic CD1d molecule. The NKT cells play a pivotal role in the orchestration of antitumor immune responses by mediating adjuvant effects that activate various antitumor effector cells of both innate and adaptive immune systems and also aid in establishing a long-term memory response. Here, we established NKT cell-targeted therapy using a newly discovered NKT cell glycolipid ligand, RK, which has a stronger capacity to stimulate both human and mouse NKT cells compared to previous NKT cell ligand. Moreover, RK mediates strong adjuvant effects in activating various effector cell types and establishes long-term memory responses, resulting in the continuous attack on the tumor that confers long-lasting and potent antitumor effects. Since the NKT cell ligand presented by the monomorphic CD1d can be used for all humans irrespective of HLA types, and also because NKT cell-targeted therapy does not directly target tumor cells, this therapy can potentially be applied to all cancer patients and any tumor types.

## Introduction

Immunotherapy, which acts by harnessing the power of patient’s own immune system, has recently emerged as a treatment option to combat cancer in addition to conventional treatment options such as surgery, chemotherapy, and radiation therapy (1). Despite encouraging results, tumor relapse and metastasis still remain a major problem for any of the current anticancer therapies. A common and significant limitation of current anticancer immunotherapies is that they often target only one type of antitumor effector cell. For example, in the immunotherapy approaches using tumor peptide-specific CTL, dendritic cells (DCs), engineered CAR-T cells, tumor-infiltrating lymphocytes, or antibodies against PD-1, the target is the effector T cell, which kills MHC-positive, but not MHC-negative, tumor cells, resulting in recurrence of MHC-negative tumor cells (2). Similarly, in the case of lymphokine-activated NK cells or NK cells generated by the enforced expression of NK receptor ligands, such as Rae1/H60/Mult-1 (NKG2D-L), the effector cells eliminate only MHC-negative tumor cells, resulting in the relapse by MHC-positive tumor cells (3). Moreover, tumor cells often undergo mutational changes that render them resistant to these therapies.

In contrast to the current immunotherapy approaches described above, natural killer T (NKT) cells (4–6), but not other effector cell types, have the potential to simultaneously activate various effector cell types, including both CD8 T and NK cells that, in turn, eliminate both MHC-positive and MHC-negative tumor cells (7). In addition, activated NKT cells can interact with immature DCs in the presence of their agonist ligand and induce maturation of DCs, thereby overcoming of the immunodeficiency status often seen in cancer patients, and also in establishment of long-term antitumor immunity (8). Therefore, NKT cell-targeted therapy is thought to be an ideal treatment approach for combating cancer and preventing tumor relapse and metastasis. Moreover, as an NKT cell ligand is presented by the monomorphic CD1d molecule (9), the ligand itself could be used as a drug that could be administered to any patient, no matter what their HLA haplotype. As the antitumor effect of NKT cell-targeted immunotherapy largely depends on activating other innate and adaptive immune cells of patient’s own immune system, which theoretically contains clones of tumor-specific effector cells that, however, cannot be effectively activated due to the immunosuppressive mechanisms mediated by tumor cells, the NKT cell-targeted therapy could be used to harness the immune system to fight any tumors type.

Based on exceptional results from preclinical studies using the potent NKT cell agonistic glycolipid ligand α-galactosylceramide (GC) (10–14), NKT cell-targeted cancer therapy in human clinical trials was started in patients with advanced or recurrent stages of various cancers. The results from these clinical trials were fairly promising, e.g., prolonged median survival time (MST) of 18.6 months in all treated patients (17 cases) with advanced non-small lung cancer refractory to the conventional therapies such as chemotherapy, radiation or surgery upon treatment with GC-pulsed peripheral blood mononuclear cells (PBMCs) compared to the MST of 4.6 months in the best supportive care patient group (15, 16). However, there is still a need for further improvements aimed to increase the efficacy of NKT cell-targeted immunotherapy. It is also important to mention that the efficacy of this NKT cell therapy is almost equivalent to that using checkpoint inhibitor such as a PD-1 mAb treatment, where the reported MST of advanced non-squamous non-small-cell lung cancer patients was 12.2 months (17).

One of approaches aimed to increase the efficacy of NKT cell-targeted antitumor immunotherapy is the optimization of NKT cell-activating ligands (18). The antitumor effect of NKT cell-based immunotherapy depends primarily on potent secretion of IFN-γ, a cytokine that actually mediates the activation of downstream cellular networks, resulting in a strong adjuvant action *via* NK cells, CD8 cytotoxic T cells, and other cell types (19), and also establishment of long-term memory responses (8). Thus, the search for a ligand capable of stimulating human NKT cells with a strong T_H_1 cytokine profile is an important objective.

In this study, we developed NKT cell-targeted cancer therapy using a newly synthesized glycolipid, termed RK, which is recognized by both mouse and human NKT cells, thereby resulting in the superior antitumor responses compared to GC. In addition, RK shows stronger activity in inducing IFN-γ release from both human and mouse NKT cells compared with the prototypical ligand GC when presented by DCs. We also demonstrate that RK-pulsed DCs have remarkable potential for induction of NKT cell-mediated adjuvant activity by activating downstream cell types such as NK and CD8 T cells, and in the establishment of long-term memory responses against a model antigen ovalbumin. Taken together, we believe that RK has a potential use in human translational studies in anticancer immunotherapy applications targeting NKT cells.

## Materials and Methods

### Human Samples and Animal Studies

All experiments involving human samples were performed with authorization from the Institutional Review Board for Human Research at RIKEN IMS. Umbilical cord blood samples were obtained from RIKEN BRC Cord Blood Bank collected with written informed consent. PBMCs from healthy donors were purchased from Astarte Biologics, LLC (USA).

### Mice

Wild-type (WT) C57BL/6 (B6) mice were purchased from Charles River Laboratories; B6.CD45.1 mice were from The Jackson Laboratory; the new *Traj18^−/−^* mice expressing undisturbed TCRα chain repertoire, except for Jα18, on B6 background were described (20). Mice were maintained in the animal facility of RIKEN IMS under specific pathogen-free conditions and were used at 8–10 weeks of age. All animal experiments were approved by RIKEN Animal Care and Use Committee.

### Neoglycolipid

The structure and the synthesis method of RK were described previously (21). In brief, reduction of an azide prepared by modification of the 6-hydroxy group of the known alcohol (2*S*,3*R*,4*E*)-2-Azido-3,4-di-*O*-benzyl-1-*O*-[2,3,4-tri-*O*-benzyl-6-*O*-(*tert*-butyldimethylsilyl)-d-galactopyranosyl]octadecane-1,3,4-triol by using Staudinger reaction gave an amine (22). The amine was acylated with cerotyl chloride to give an amide. Deprotection of all of the benzyl groups of the amide by hydrogenolysis afforded RK as white powder.

### Cell-Free Antigen Presentation Assay

Flat bottom 96-well culture plates were coated with 2 µg of soluble dimeric mouse CD1d:Ig fusion protein (BD Biosciences) in 50 µL PBS. After incubation for 6 h at 4°C, 50 µl of the indicated lipid antigens diluted in PBS were added, and the plate was incubated overnight at 37°C. The next day, the wells were washed with PBS and incubated with complete culture medium before the addition of the NKT cell hybridoma 2E10 at 1 × 10^5^ cells per well. Culture supernatants were collected after 16 h for the cytokine measurement assay by ELISA.

### Cell Preparation and Flow Cytometry

Single-cells from designated mouse organs and mononuclear cells from umbilical cord blood samples were prepared as described previously (23, 24). Surface antigen staining was performed after Fc receptor blocking using TruStain fcX™ or human TruStain FcX™ (BioLegend). Forward light-scatter gating and 7-AAD staining (BD Biosciences) were used to gate out doublets and dead cells. Samples were acquired on FACS Canto II (BD Biosciences), and data were analyzed with FlowJo 10.0.8r1 software (Tree Star). Anti-mouse antibodies were as follows: CD3ε^+^ or -PE (145-2C11), CD8α^+^ or -PE (53-6.7), CD19-PerCP-Cy5.5 (1D3), CD45.1-FITC (A20), CD45.2-PE (104), NK1.1-APC (PK136), and TCRβ^+^ (H57-597). Anti-human antibodies were as follows: TCR Vα24-Jα18-Brilliant Violet 421 (6B11), CD3-FITC, or -APC (OKT3). Above mAbs were from BD Biosciences or BioLegend. Vehicle-, RK-, or GC-loaded soluble dimeric mouse or human CD1d:Ig fusion protein (BD Biosciences) were used with APC-anti-mouse IgG1 (X56; BD Biosciences) to detect mouse or human NKT cells, respectively (23).

### Intracellular Cytokine Staining

Splenocytes were seeded at 2 × 10^7^ cells/mL in a complete medium supplemented with GolgiPlug (BD Biosciences) and cultured for 1 h at 37°C. After cell surface staining, cells were fixed, permeabilized and stained with PE-labeled anti-IFN-γ (XMG1.2) from BioLegend using BD Cytofix/Cytoperm™ Fixation/Permeabilization Kit (BD Biosciences) according to the manufacturer’s instructions.

### *In Vitro* Cell Culture Conditions

The NKT cell hybridoma 2E10 was cultured as described (25). Bone marrow-derived DCs from B6 mice were prepared as described (23, 26), where after 6 days of culture in a complete RPMI-1640 medium (ThermoFisher Scientific) supplemented with 5 ng/mL mGM-CSF (R&D), DCs were purified with AutoMACS and anti-mouse CD11c microbeads (Miltenyi Biotec). Human DCs were prepared as described (27), where CD14^+^ monocytes were purified from PBMNCs with a MACS LS column and anti-human CD14 microbeads (Miltenyi Biotec) and cultured for 6 days in a DendriMACS GMP medium containing 800 U/mL hGM-CSF and 250 U/mL hIL-4 (all from Miltenyi Biotec). Human umbilical cord blood derived mononuclear cells were prepared by density gradient centrifugation using Ficoll-Paque Plus (GE Healthcare), and NKT cell cultures were performed as reported (23) with a minor modification, where the culture medium consisted of 50% AIM-V medium (ThermoFisher Scientific), 45% RPMI-1640, 5% heat-inactivated fetal bovine sera (Sigma), 1 × NEAA, 1 mM sodium pyruvate, 55 µM 2-ME, 2 mM l-glutamine, and 100 U/mL penicillin/streptomycin (all from ThermoFisher Scientific) and supplemented with 100 U/mL hIL-2 (Shionogi, Japan).

### CD40 Ligation and Real-time Quantitative PCR

Human PBMNC-derived DCs (1 × 10^5^ per well) were cultured in the presence or absence of histidine-tagged recombinant human CD40 Ligand (0.1 µg/mL; from R&D) and His Tag Antibody (10 µg/mL; from R&D) in 96-well culture plates for 12 h. RNA was purified using an RNeasy Plus Micro kit (Qiagen), and cDNA was prepared with Superscript VILO cDNA Synthesis Kit (Life Technologies). Quantitative real-time PCR was performed with the ABI PRISM 7900HT system (Applied Biosystems) using FastStart Universal Probe Master Mix (Roche). Relative gene expression was calculated with the 2^−ΔΔCt^ method, where the *GAPDH* expression level served as an internal control. Taqman^®^ Gene Expression Assays for *IL12B* (Hs01011518_m1) and *GAPDH* (Hs02758991_g1) were from Applied Biosystems.

### Cytokine Measurements

IFN-γ concentrations in plasma or culture supernatants were quantified with ELISA kits for mouse IFN-γ (R&D) or human IFN-γ (BD Biosciences). The levels of mouse IL-12p70, mouse IL-4, and human IL-4 were measured with a cytometric bead array (CBA) (BD Biosciences).

### Induction of T Cell-Mediated Immunity against OVA Antigen

To identify OVA-specific T cells expanded upon NKT stimulation *in vivo*, splenocytes were prepared according to a published report (28) with some minor modifications. In brief, splenocytes (2 × 10^7^ cells per mouse) pulsed with OVA peptide (Worthington Biochemical) were administered intravenously together with 5 × 10^4^ DCs that had been pulsed overnight with RK or GC at 100 ng/mL. The mice were sacrificed after 7 days, and liver mononuclear cells were stained for the presence of OVA-specific CD8 T cells using T-Select H-2Kb OVA Tetramer-SIINFEKL-APC (MBL). For the experiments to assess the induction of long-term OVA-specific immunity, OVA-pulsed, osmotically shocked splenocytes (2 × 10^7^ cells per mouse) were intravenously injected together with unpulsed or RK-pulsed DCs (1 × 10^6^ cells per mouse). These mice received another injection of unpulsed or RK-pulsed DCs (1 × 10^6^ cells per mouse) 4 days after the initial immunization. At the indicated time points, mice were sacrificed and splenocytes were directly assessed for the presence of OVA-specific CD8 T cells using T-Select H-2Kb OVA Tetramer-SIINFEKL-APC (MBL), or were primed *in vitro* with or without 1 µM SIINFEKL (OVA_257–264_) peptide (Abbiotec) for 6 h in the presence of GolgiPlug (BD Biosciences). The cells were then stained for cell surface markers, fixed with the Cytofix/Cytoperm Plus permeabilization kit (BD Biosciences), and stained with PE-labeled anti-IFN-γ (XMG1.2) from BioLegend.

### B16 Melanoma Metastasis Model

Mice were anesthetized and the spleen was surgically removed on day 0 after intrasplenic inoculation of B16 melanoma (5 × 10^5^) cells. Four days after the B16 inoculation, mice were injected intravenously with either RK- or GC-pulsed bone marrow-derived DCs (3 × 10^4^ or 1 × 10^5^). The mice were sacrificed after 2 weeks, and the liver was visually or quantitatively evaluated for B16 metastases.

### Measurement of B16 Melanoma Metastases in a Liver Tissue with Visible Light Absorption Spectrophotometry

Liver tissues were homogenized in 10 mL of a sodium hydroxide (1 M) solution with IKA Ultra-Turrax tube disperser workstation system using DT-20 dispersing tube with rotor–stator for 30 s. Then 1 mL of homogenate was heated at 75°C for 1.5 h, diluted further with sodium hydroxide, and 0.2 mL of diluted homogenate was used for measurement of absorbance at 405 nm with a Wallace 1420 ARVO MX multi-label plate reader (PerkinElmer). A standard curve was generated using serially diluted B16 melanoma cells, and normalized absorbance values were calculated relative to the B16 only (untreated) controls.

### Statistical Analysis

Where indicated, two-tailed unpaired *t* tests were done using PRISM 6 software (GraphPad). *P* < 0.05 was considered statistically significant.

## Results

### RK Is Recognized by Mouse NKT Cells and Elicits Strong IFN-γ Secretion

**Figure 1:**
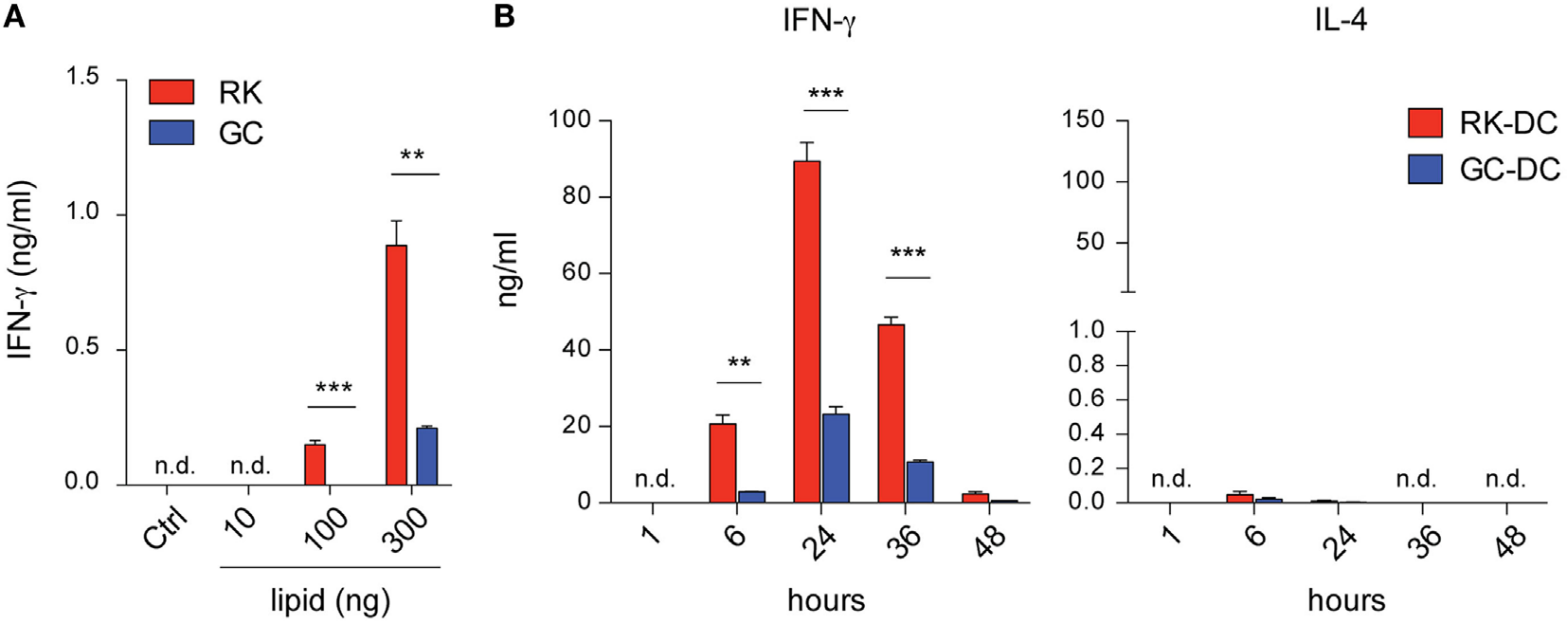
IFN-γ release from mouse Vα14^+^ natural killer T (NKT) cells activated with RK or galactosylceramide (GC) glycolipids. **(A)** NKT cell hybridoma E210 cells were plated 1 × 10^5^ cells per well into 96-well culture plates, which were previously coated with soluble CD1d and incubated with RK or GC at the indicated concentrations overnight. Vehicle was used as a control in the NKT cell hybridoma activation assay. The culture supernatants were collected after 16 h and IFN-γ levels were measured by ELISA. Data are mean ± SEM from triplicate wells. The data are representative from two independent experiments with similar results. **(B)** *In vivo* antigen presentation assay with RK- or GC-pulsed dendritic cells (DCs). B6 mice were injected intravenously with 5 × 10^5^ RK- or GC-pulsed DCs per mouse, and levels of IFN-γ and IL-4 in the sera collected at the indicated time points were measured by ELISA or cytometric bead array, respectively. Data are mean ± SEM from three mice and repeated three times with similar results. **P < 0.01; ***P < 0.001, unpaired Student’s *t*-test; n.d., not detected.

In search of a potent NKT cell-activating antigen with superior antitumor characteristics compared to the widely used glycolipid ligand α-GC, we have newly synthesized a series (172) of neoglycolipids and screened these compounds for their potential to activate NKT cells. For the initial screening assay to measure the NKT cell activating potential of the newly synthesized compounds, we used a cell-free antigen presentation assay, in which soluble CD1d was coated on a culture plate, pulsed with glycolipid antigens, and IFN-γ release from the NKT cell hybridoma 2E10 expressing an invariant Vα14Jα18 TCRα paired with Vβ8 was used as a readout (25). Results showed that one of the newly synthesized glycolipid, termed RK, is a much more potent activator of the NKT cell hybridoma than GC in a glycolipid antigen dose-dependent manner (Figure 1A).

Next, we sought to verify and extend the *in vitro* screening result with a more physiologically relevant *in vivo* antigen presentation assay, where WT B6 mice were intravenously injected with RK-pulsed mouse bone marrow-derived dendritic cells (RK-pulsed DCs). Compared with GC-pulsed DCs, the RK-pulsed DCs showed a significantly higher (>4-fold) ability to elicit IFN-γ secretion in the sera collected at both early (6 h) and later (24, 36 h) time points. Conversely, the serum levels of IL-4 were similarly lower in mice injected with RK-pulsed or GC-pulsed DCs compared to IFN-γ levels (Figure 1B). In addition, as expected, IFN-γ was not detected in the sera of NKT cell-deficient *Traj18^−/−^* mice, verifying the NKT cell specificity of both RK- and GC-pulsed DCs (data not shown). These results demonstrated that RK-pulsed DCs induce stronger T_H_1-type cytokine secretion compared with GC-pulsed DCs.

### RK Is Recognized by Human NKT Cells and Elicits Strong IFN-γ Secretion

**Figure 2:**
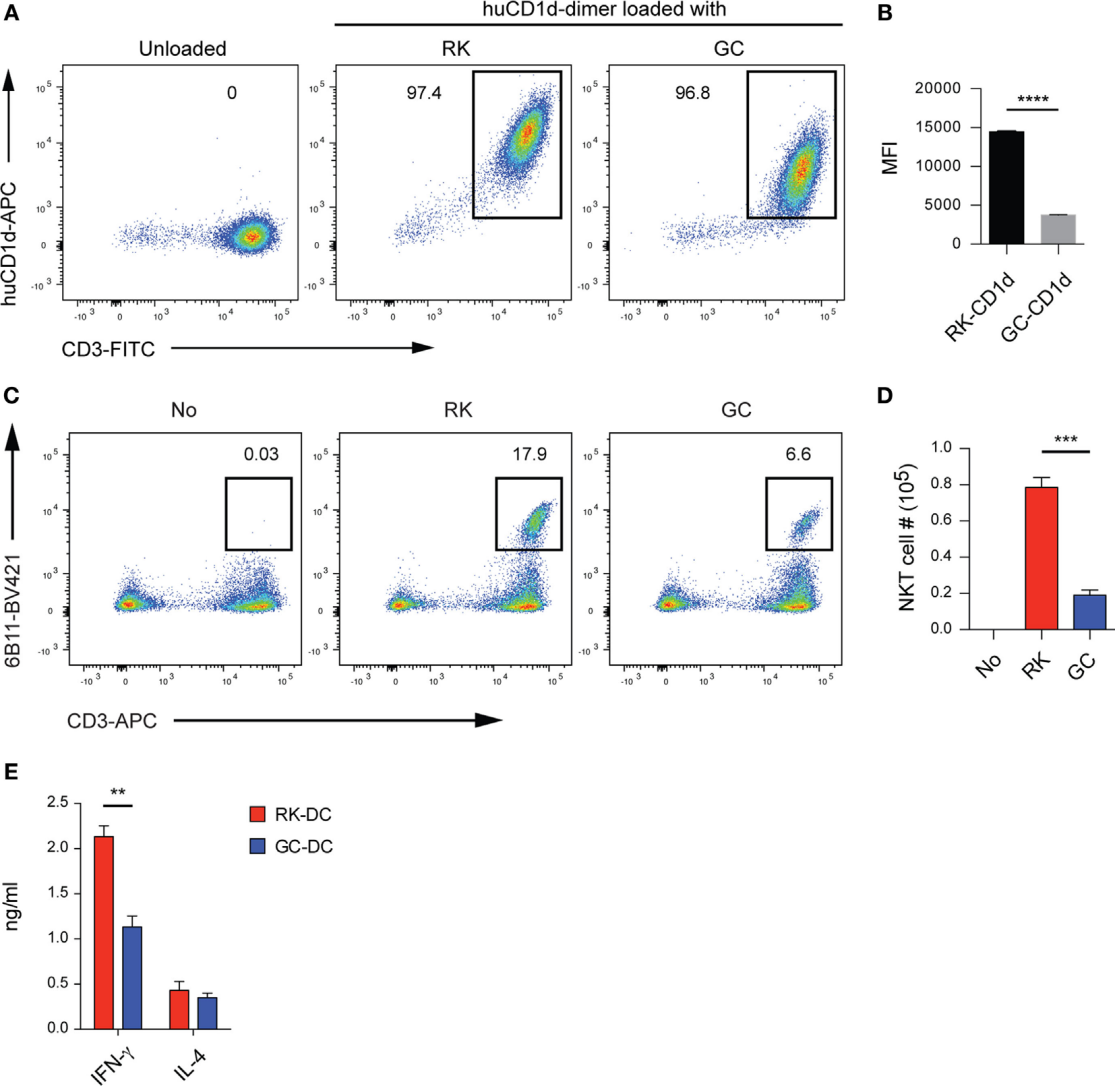
The novel glycolipid RK is recognized by human natural killer T (NKT) cells. **(A)** Staining of an NKT-iPSC line with RK- or galactosylceramide (GC)-loaded human CD1d dimers. Flow cytometry plots are representative from triplicate samples per group. Numbers indicate the percentage of human CD1d dimer^+^CD3^+^ cells within the 7-AAD^−^ viable lymphocyte gates. **(B)** The mean fluorescence intensity (MFI) levels of human CD1d dimers loaded with RK- or GC, which were gated as shown in panel **(A)**. Data are mean ± SEM from triplicate samples per group. Experiments were repeated three times with similar results. **(C)** Human NKT cell expansion upon culturing with RK. Umbilical cord blood mononuclear cells were cultured in the presence or absence of the indicated glycolipids (100 ng/mL) for 19 days in complete media supplemented with 100 U/mL hIL-2. The cultures were re-stimulated with fresh glycolipid-pulsed antigen-presenting cells on culture day 9. Numbers on flow cytometry plots show frequencies (mean ± SEM, *n* = 3 samples per group) of Vα24^+^CD3^+^ human NKT cells within gated viable lymphocytes. **(D)** Absolute NKT cell numbers (mean ± SEM, *n* = 3 samples per group) of Vα24^+^CD3^+^ human NKT cells as shown in panel **(C)**. Experiments were repeated with two different donors with similar results. **(E)** IFN-γ and IL-4 release from human NKT cells activated with RK-pulsed dendritic cells (DCs). Human NKT cells (5 × 10^4^ per well) were co-cultured for 48 h with the same numbers of peripheral blood monocyte-derived DCs that were pulsed overnight with RK or GC at 100 ng/mL. IFN-γ and IL-4 levels in culture supernatants were measured with ELISA or cytometric bead array, respectively. Data are mean ± SEM from triplicate wells. Experiments were repeated three times with similar results. **P < 0.01; ***P < 0.001; ****P < 0.0001, unpaired Student’s *t*-test.

Because of its importance for therapeutic application, we wished to extend our mouse experiments into a human system to assess whether the RK antigen is recognized by human NKT cells. To this end, we first loaded human CD1d dimers with RK or GC according to the previously published method (23), and stained a human NKT-iPSC line described previously (29). Flow cytometry results clearly demonstrated that RK-loaded human CD1d molecules efficiently stain human NKT cells, where GC-loaded CD1d and unloaded CD1d were used as positive and negative staining controls, respectively (Figure 2A). Of note, the mean fluorescence intensity (MFI) of RK-loaded CD1d was significantly higher than GC-loaded CD1d (Figure 2B), which mirrored results obtained by staining of mouse NKT cells with mouse RK-loaded CD1d (Figures S1A–C in Supplementary Material). The elevated MFI levels could be best explained by the higher affinity of the RK-loaded CD1d complex for the invariant TCR of NKT cells.

Next, we examined whether RK can activate human NKT cells using proliferative responses upon *in vitro* culture as the readout. To this end, we cultured umbilical cord derived mononuclear cells in the presence of RK or GC for a total of 19 days. Flow cytometry analyses demonstrated that RK induces significantly higher expansion of human NKT cells compared with GC (Figures 2C,D). These results indicate that RK not only strongly stimulates human NKT cells but also is efficiently processed and presented by human antigen-presenting cells (APCs).

To investigate whether RK presented by human DCs has the capacity to induce increased T_H_1 cytokine release from human NKT cells, we carried out an antigen presentation assay using peripheral blood monocyte-derived DCs as APCs together with a human NKT cell line as effector cells. The IFN-γ but not IL-4 levels in 48 h co-culture supernatants were significantly higher in RK-pulsed DCs compared with GC-pulsed DCs (Figure 2E).

In summary, these results demonstrate that RK is efficiently presented by human DCs and that these RK-pulsed DCs show superior activity compared to GC-pulsed DCs in promoting T_H_1 cytokine production from human NKT cells.

### The *In Vivo* Dynamics of RK-Pulsed DCs

**Figure 3:**
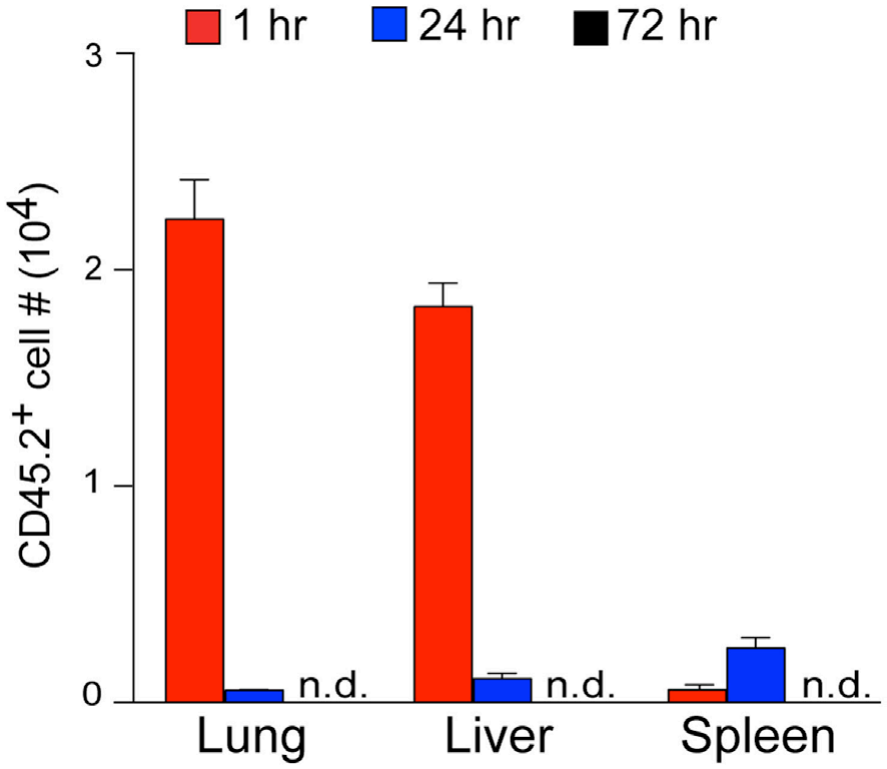
The *in vivo* tissue dynamics of RK-pulsed dendritic cells (DCs). RK-pulsed DCs prepared from B6.CD45.2 donors (1 × 10^7^) were adoptively transferred *via* the tail veins of B6.CD45.1 congenic recipient mice, and CD45.2^+^ DCs of donor origin were assessed by flow cytometry at 1, 24, and 72 h time points after injection. Absolute numbers of CD45.2^+^ donor-derived cells found within the indicated organs are shown (mean numbers ± SEM, *n* = 3 recipient mice). Experiments were repeated two times with similar results; n.d., not detected.

It is well known that GC-pulsed DCs mediate strong antitumor activities upon systemic administration (14, 20). For pharmacokinetic characterization of RK-pulsed DCs, we investigated their tissue distribution and dynamics. To this end, we prepared RK-pulsed DCs from B6.CD45.2 donors and then injected these cells intravenously into B6.CD45.1 congenic recipient mice. CD45.2^+^ DCs of donor origin were enumerated at 1, 24, and 72 h time points after injection. Flow cytometry analyses of recipient mice demonstrated that injected DCs of donor origin are eliminated within 72 h when administered *via* the intravenous route (Figure 3). The distribution pattern of donor DCs suggests that the majority are located in the lung and liver at the 1-h time point and to a lesser extent in the spleen. The number of donor DCs gradually decreased until they were no longer detectable at the 72-h time point. These results imply that the RK-pulsed DCs are quickly eliminated from the organism, soon after these cells have done their job to activate NKT cells.

### Adjuvant Effects on Immune Cell Types upon RK-Pulsed DC Administration

**Figure 4:**
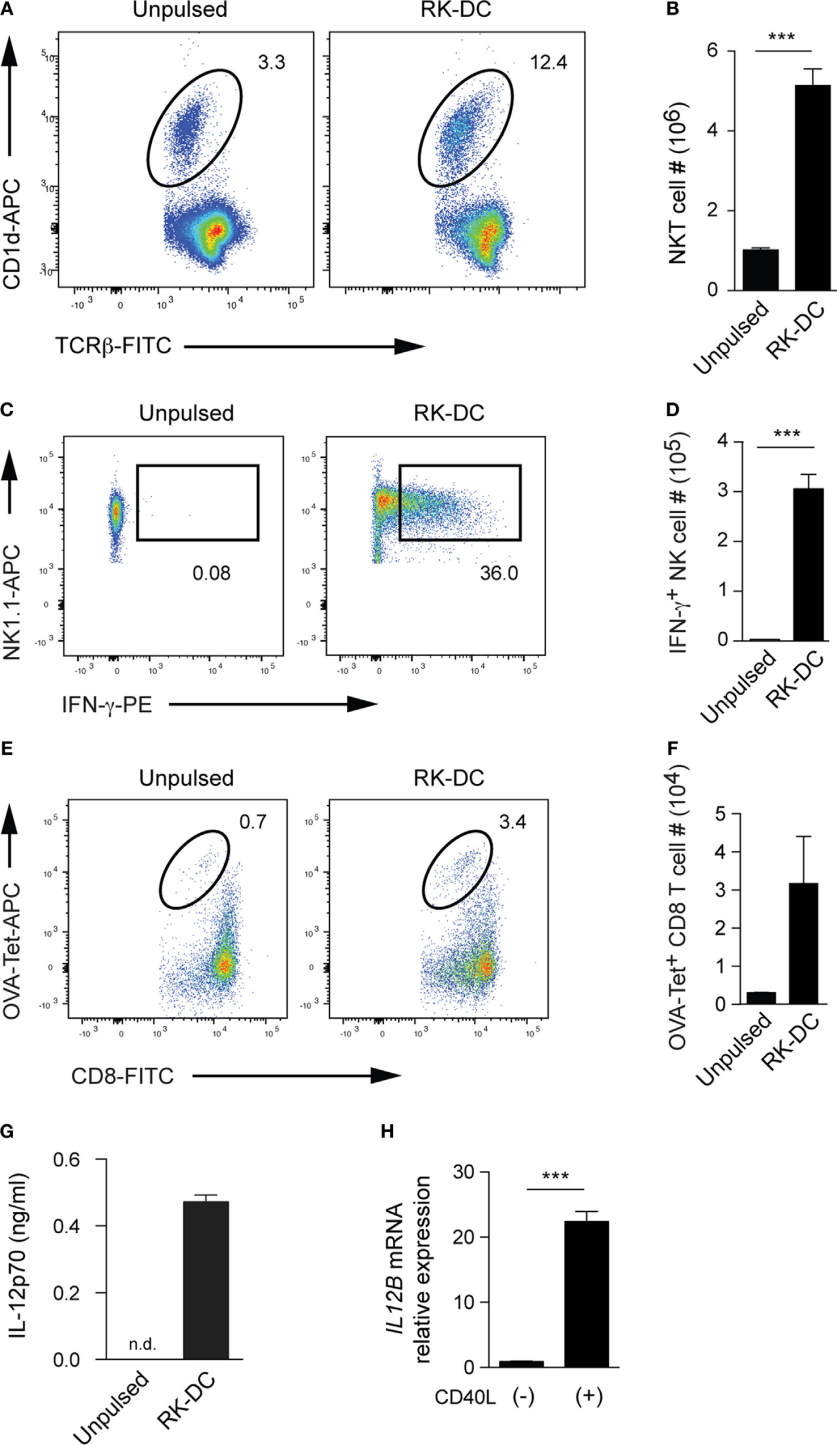
The *in vivo* adjuvant effects of RK-pulsed dendritic cells (DCs). **(A)** *In vivo* expansion of natural killer T (NKT) cells upon injection of RK-DCs. Flow cytometry of wild-type B6 splenocytes 6 days after intravenous injection of 5 × 10^4^ unpulsed DCs or RK-DCs. Numbers indicate percentages of CD1d dimer^+^TCRβ^+^ NKT cells within the 7-AAD^−^CD8^−^CD19^−^ viable splenocyte gate. Data are representative of 3 mice per group. **(B)** Absolute numbers of splenic NKT cells as shown in panel **(A)**. Data are mean ± SEM from three mice per group. **(C)** Intracellular analysis of IFN-γ production by splenic NK cells. Unpulsed- or RK-pulsed DCs (5 × 10^4^ per mouse) were injected intravenously into B6 mice. Splenocytes were isolated after 16 h and cultured in complete media containing GolgiPlug (BD Biosciences) for 1 h. Numbers on flow cytometry plots show percentages of NK1.1^+^IFN-γ^+^ cells among NK1.1^+^CD3ε^−^ gated cells. The data are representative of *n* = 3 mice per group. **(D)** Absolute cell numbers of IFN-γ^+^ NK cells. Data are mean ± SEM from *n* = 3 mice per group. **(E)** Detection of OVA-specific tetramer-positive CD8 effector T cells. B6 mice were immunized with OVA antigen together with unpulsed- or RK-pulsed DCs (5 × 10^4^ per mouse) on day 0 and liver mononuclear cells were analyzed 7 days later. Numbers on flow cytometry plots indicate percentages of OVA tetramer-positive cells among viable CD8 T cells. **(F)** Absolute numbers of OVA tetramer + CD8 T cells (mean ± SEM, *n* = 3 mice per group) gated as shown in panel **(E)**. Experiments shown in panels **(A–F)** were repeated two times with similar results. **(G)** Detection of IL-12p70 in the sera collected 6 h after intravenous injection of 5 × 10^4^ unpulsed- or RK-pulsed DCs into B6 mice. Serum IL-12p70 levels were measured by cytometric bead array. Data are mean ± SEM from three mice, and experiments were repeated three times with similar results. **(H)** Detection of *IL12B* mRNA encoding IL-12p40 by real-time quantitative PCR. RK-pulsed human PBMNC-derived DCs (1 × 10^5^ per well) were cultured for 12 h with or without recombinant human CD40 ligand. Bars depict the relative gene expression (mean ± SEM from triplicate wells) with *GAPDH* used as internal control. The experiment was repeated with three different donors with essentially similar results. ***P < 0.001, unpaired Student’s *t*-test; n.d., not detected.

We went on to investigate *in vivo* adjuvant effects of RK-pulsed DCs on both innate and adaptive immune cell types, such as NKT, NK, and CD8 T cells of the host. Flow cytometry analyses of splenocytes from B6 mice on day 6 post-injection revealed that both the frequency and absolute number of splenic NKT cells was clearly augmented upon RK-pulsed DC administration compared with control mice injected with unpulsed DCs (Figures 4A,B). These results suggest that adoptive transfer of RK-pulsed DCs induces a robust expansion of NKT cells *in vivo*.

To directly assess the NKT cell-mediated trans-activation of NK cells (30, 31), we intravenously injected RK-pulsed DCs and then investigated IFN-γ production by splenic NK cells at 16 h post-injection. Flow cytometry analysis of splenic NK cells, which were detected as NK1.1^+^CD3ε^−^ cells, revealed that frequency and number of IFN-γ^+^ NK cells from RK-pulsed DC-injected animals were significantly increased compared to mice injected with unpulsed DCs (Figures 4C,D). These data suggest that RK-pulsed DCs induce strong trans-activation of NK cells, which in turn leads to the long-lasting IFN-γ secretion.

To directly assess the NKT cell-mediated adjuvant activity on adaptive immune responses, we immunized B6 mice with OVA antigen together with unpulsed or RK-pulsed DCs and analyzed liver mononuclear cells 7 days later for the presence of OVA-specific CD8 T cells. Flow cytometry analysis showed that the RK-pulsed DC injection resulted in greatly increased frequencies and numbers of OVA tetramer-positive CD8 T cells compared with those of unpulsed DCs (Figures 4E,F). These results indicate that RK-pulsed DCs show strong adjuvant effects in the induction of antigen-specific immunity.

Then, we wished to test whether *in vivo* administration of RK-pulsed DCs results in IL-12 release, which in turn provides a positive feedback effect on IFN-γ production from NK and NKT cells (32–34). To this end, we assessed sera samples collected 6 h after injection with RK-pulsed DCs or unpulsed DCs. The CBA results clearly revealed strong IL-12p70 release from mice injected with RK-pulsed DCs, while no IL-12p70 was detected in mice that received unpulsed DCs (Figure 4G). Given that the mechanism of IL-12 release from DCs is thought to be a consequence of NKT cell-mediated maturation of glycolipid-presenting immature DCs through CD40/CD40L interactions (35), we wished to further extend these results using human PBMNC-derived DCs pulsed with RK and assess the IL-12 induction mimicking the CD40/CD40L interaction *in vitro*. Real-time PCR analyses of human RK-pulsed DCs cultured with or without CD40 ligand clearly demonstrated a significant induction of *IL12B* mRNA encoding IL-12p40 upon CD40 ligation (Figure 4H).

Taken together, these results suggest a scenario where adoptively transferred RK-pulsed DCs, in addition to eliciting strong IFN-γ production by NKT cells, mediate positive feedback to further enhance IFN-γ production through their ability to release IL-12 upon interaction with endogenous NKT cells of the host through CD40/CD40L. These cellular interactions presumably lead to observed remarkably strong adjuvant effects, resulting in the activation of players associated with innate and adaptive immune systems such as NK cells and antigen-specific effector CD8 T cells.

### RK-Pulsed DCs Promote an Establishment of Long-term T Cell Memory Responses

**Figure 5:**
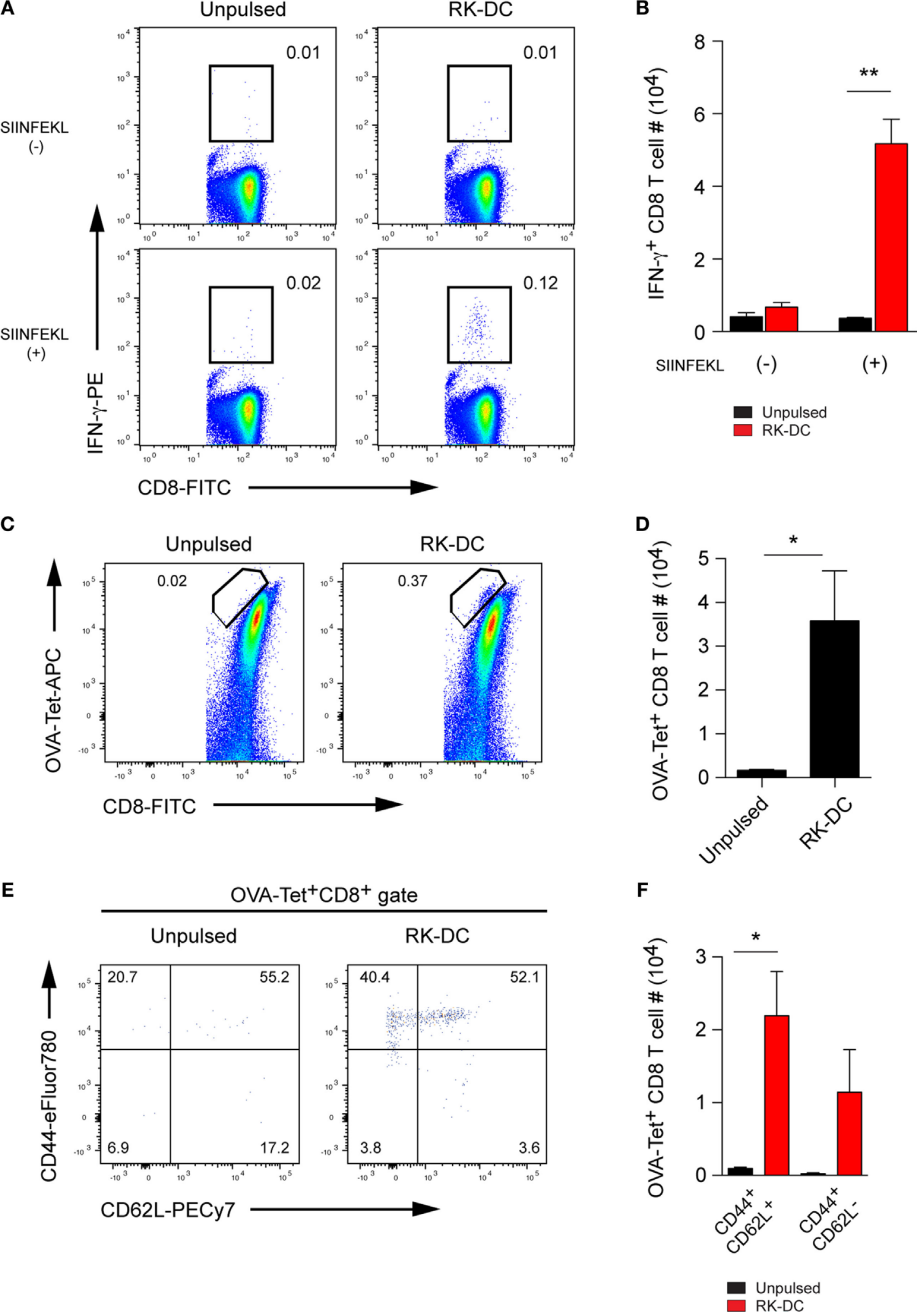
Evaluation of long-term T cell memory responses. **(A)** Detection of IFN-γ-producing memory CD8 T cells. B6 mice were immunized with OVA antigen and injected with unpulsed- or RK-pulsed dendritic cells (DCs) (1 × 10^6^ cells per injection) on day 0 and day 4 by the intravenous route. Splenocytes were harvested 3 months later and were activated *in vitro* with or without SIINFEKL (OVA_257–264_) peptide for 6 h in the presence of GolgiPlug Protein Transport Inhibitor. Cells were stained with anti-IFN-γ mAb using a Cytofix/Cytoperm intracellular staining kit. Numbers on flow cytometry plots indicate frequencies of IFN-γ producing CD8 T cells gated as CD3ε^+^CD8^+^ lymphocytes. Data are representative from three mice per group. **(B)** Absolute numbers of IFN-γ^+^ CD8 T cells (mean ± SEM, *n* = 3 mice per group) gated as shown in panel **(A)**. **(C)** Frequencies and **(D)** absolute numbers (mean ± SEM, *n* = 3 mice per group) of OVA-specific memory CD8 T cells detected 9 months after immunization. OVA immunization and adoptive transfer of unpulsed- or RK-pulsed DCs were done as described in panel **(A)**. Numbers on flow cytometry plots show percentages of OVA tetramer^+^CD8^+^ memory T cells among CD3ε^+^CD8^+^ gated splenocytes. **(E)** Frequencies and **(F)** numbers (mean ± SEM, *n* = 3 mice per group) of CD44^+^CD62L^+^ central memory and CD44^+^CD62L^−^ effector memory antigen-specific CD8 T cells among gated OVA tetramer^+^CD8^+^ T cells analyzed 9 months after immunization as shown in panel **(C)**. All experiments were repeated two times with similar results. *P < 0.05; **P < 0.01, unpaired Student’s *t*-test.

It was previously reported that NKT cells were required for the establishment of long-term protective immunity against several tumor cell lines that lasted even after one year of vaccination with GC-loaded tumor cells used as vaccine delivery vectors (8). Here, we investigated whether NKT activation with RK-pulsed DCs can induce long-term antigen-specific T cell-mediated memory responses. To test this, we made use of experimental model with OVA as an artificial antigen, where B6 mice were immunized with osmotically shocked splenocytes pulsed with OVA together with unpulsed or RK-pulsed DCs by the intravenous route. When we assessed the immunized mice 3 months later, the *in vitro* functional assay results clearly demonstrated the presence of clonotypically expanded IFN-γ producing OVA-specific CD8 T cells in mice that had received RK-pulsed DCs, whereas these OVA-specific CD8 T cells were virtually undetectable in mice that had received unpulsed DCs (Figures 5A,B). When we assessed similarly immunized mice after 9 months, we could still detect significant numbers of OVA-specific T cells (Figures 5C,D), where the numbers of both CD44^+^CD62L^+^ central memory and CD44^+^CD62L^−^ effector memory OVA tetramer^+^ CD8 T cells were expanded in mice treated with RK-pulsed DCs compared with those treated with unpulsed DCs (Figures 5E,F). These results demonstrate that RK-pulsed DCs have strong adjuvant effects that confer the induction of long-term antigen-specific T cell memory, which presumably plays a prime role in the long-lasting T cell-mediated immunity against the MHC-positive tumors.

### RK-Pulsed DCs Possess Superior Antitumor Activity

**Figure 6:**
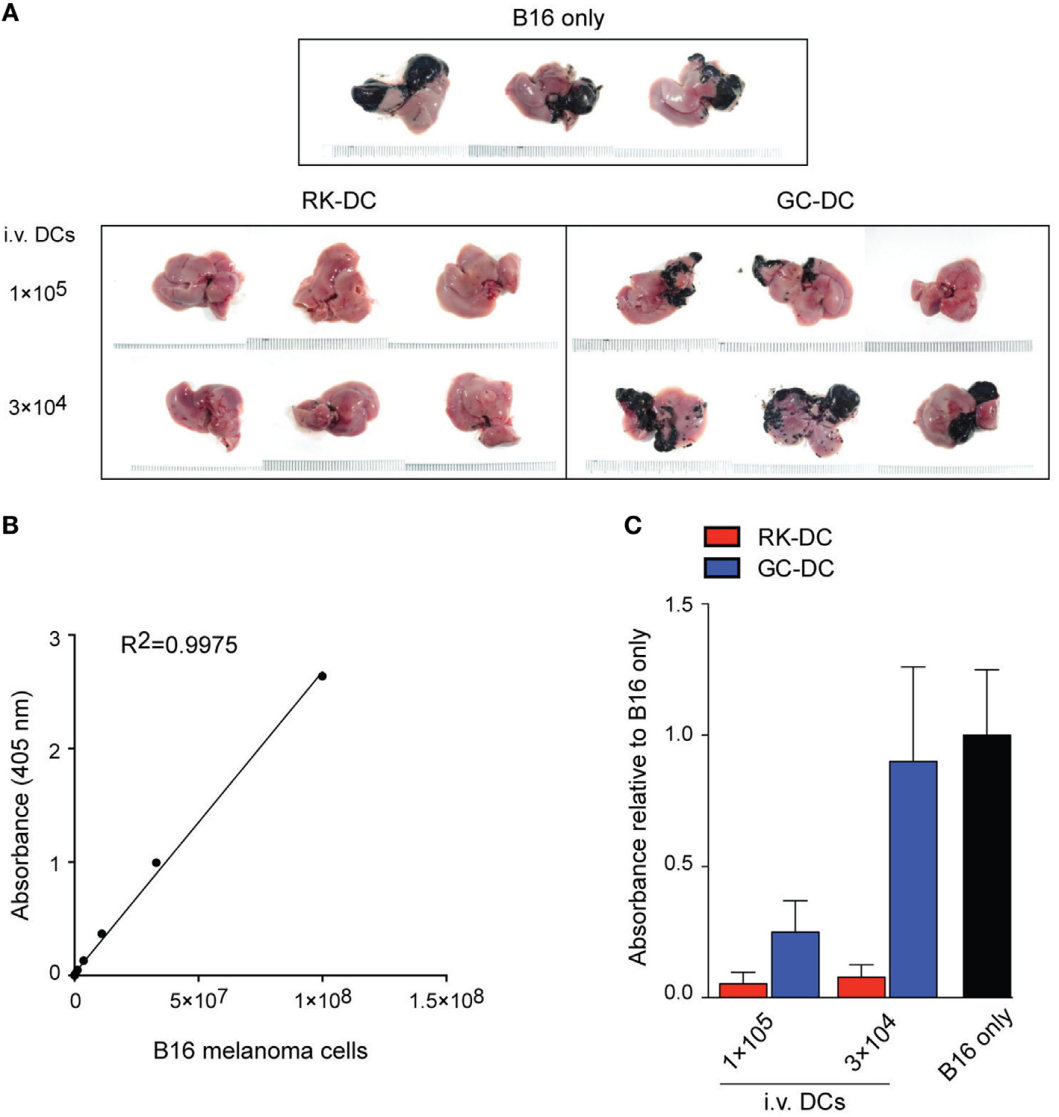
Antitumor activity of RK-pulsed dendritic cells (DCs). **(A)** Inhibition of B16 melanoma metastasis by specific activation of natural killer T cells with RK-DCs. The antitumor effect of RK-DCs was assessed using the B16 melanoma liver metastasis model. Tumor cells were inoculated into B6 mice on day 0, and the indicated numbers of RK- or galactosylceramide (GC)-pulsed DCs were injected intravenously on day 4. Untreated control mice were inoculated only with the B16 melanoma. Individual liver tissue images obtained from three mice per experimental group on day 14 post-injection are shown. **(B)** Standard curve generated by melanin measurement using visible light spectroscopy at 405 nm, obtained from serially diluted B16 melanoma cells. **(C)** Quantitative analysis of B16 melanoma burden in the liver tissues shown in panel **(A)**. Values represent calculated ratios of melanin concentrations assessed by visible light spectroscopy relative to the untreated animals inoculated only with the B16 melanoma (mean ± SEM, *n* = 5 mice per group). All experiments were repeated three times with similar results.

Since the *in vivo* biological effects of RK-pulsed DCs were compatible with their being promising antitumor immunotherapy therapeutics, we investigated the antitumor properties of RK-pulsed DCs. For this purpose, we used a B16 melanoma liver metastasis model in which GC-pulsed DCs have been shown to completely eradicate the tumor (14, 20). Therefore, in order to directly compare the antitumor potential of RK- and GC-pulsed DCs, we modified our previously reported method. We established a liver metastasis model by intrasplenic injection of B16 melanoma cells at day 0, and then after 4 days we injected RK- or GC-pulsed DCs into the melanoma-bearing hosts. Macroscopic observation of liver tissues obtained after two weeks of injection with DCs clearly showed that RK-pulsed DCs have much stronger antitumor activity compared with GC-pulsed DCs (Figure 6A). Strikingly, as few as 3 × 10^4^ RK-pulsed DCs almost completely eradicated the tumor, while the same number of GC-pulsed DCs did not show any therapeutic effect. Moreover, the effect from injection of 3 × 10^4^ RK-pulsed DCs was almost comparable to the effect from injection of 1 × 10^5^ RK-DCs. In addition to the macroscopic evaluation, we also attempted to quantitatively measure the B16 melanoma burden in the liver by homogenizing the liver tissue and analyzing the melanin concentration in samples by visible light spectroscopy, based on a standard curve obtained with serially diluted melanoma cells (Figure 6B). Results from quantitative analyses of B16 melanoma burden confirmed the results derived from macroscopic observation (Figure 6C). Collectively, these results clearly demonstrate that RK-pulsed DCs have superior antitumor activity compared with the widely used GC-pulsed DCs.

## Discussion

In this study, we have developed NKT cell-targeted cancer therapy using a newly synthesized glycolipid, termed RK, which is recognized by both mouse and human NKT cells, mediates strong adjuvant activity on various cell types in the innate and acquired immune systems, establishes long-term T cell memory responses (lasting more than 9 months), and shows superior antitumor responses.

There are several possible mechanisms that could explain this superior antitumor activity resulting from NKT cell activation with RK. One interpretation is that RK has a much stronger ability to induce IFN-γ release from both mouse and human NKT cells compared with GC when presented by professional APCs such as DCs (as shown in Figure 1B). In agreement with previous studies suggesting that the strong binding affinity of the TCR with the glycolipid/CD1d complex may lead to the T_H_1 skewed cytokine release (27, 36), the staining intensities of RK-loaded human or mouse CD1d molecules showed significantly higher MFI than those of GC-loaded CD1d. This could partly explain the augmented IFN-γ release from NKT cells, which may have resulted from higher binding affinity of the TCR/RK/CD1d triple complex. We also found that adoptive transfer of RK-pulsed DCs strongly induced IL-12 release, presumably induced upon interaction of transferred RK-pulsed DCs with endogenous NKT cells through a CD40/CD40L interaction, which further enhances IFN-γ secretion through a positive feedback mechanism.

We have also made an interesting observation that adoptive transfer of RK-pulsed DCs mediated strong adjuvant effects before the cells were eliminated from the recipient within 72 h, yet they could still activate various downstream effector cell types such as NK and antigen-specific CD8 T cells. This feature of RK-pulsed DCs to activate both NK and CD8 T effector cells and other antitumor effector cells is particularly important for the immunotherapy approach, because for an optimal anticancer therapy both MHC-positive and MHC-negative tumor cells should be eliminated simultaneously to avoid tumor relapse and metastasis. In other words, this dichotomy represents the main threat in anticancer therapy because tumors in general contain both cell types and often undergo mutational changes that allow immune evasion. Furthermore, the adjuvant activity of RK-pulsed DCs has important implications because tumor cells, unlike pathogens, are usually poorly immunogenic as they do not contain any endogenous adjuvant materials. It is this property of tumor cells that makes it difficult for the immune system to mount efficient antitumor immune responses in patients without use of adjuvants.

Another important finding was that RK-pulsed DCs have the capacity to establish long-term memory responses, where we could still detect ovalbumin-specific CD44^+^CD62L^+^ central memory CD8 T cells even after 9 months in OVA-primed mice that had received only a single injection of RK-pulsed DCs. The establishment of long-term T cell-based memory is particularly important in cancer patients to prevent tumor recurrence by conferring long-lasting tumor protection.

In summary, RK-pulsed DCs strongly activate NKT cells *in vivo*, and through their ability to secrete IL-12, which is induced upon interaction with endogenous NKT cells of the host *in situ*, augment IFN-γ release that results in strong adjuvant activity for both innate and acquired effector systems. The endpoint result of these cellular interactions is the activation of both cytotoxic CD8 T cells and NK cells that eliminate MHC-positive and MHC-negative tumors, respectively, resulting in the tumor eradication without relapse.

Our results warrant further human translational studies using the newly discovered agonist antigen RK. Moreover, RK-pulsed DCs are promising targets for future clinical application because of their potent adjuvant activities on both cytotoxic CD8 T cells and NK cells to eliminate MHC-positive and MHC-negative tumor cells, respectively, and their potential to establish long-term effector memory. Currently, we have started translational research (AMED project) on the establishment of an immunotherapy method using the novel glycolipid ligand RK.

## Ethics Statement

All experiments were performed in accordance with the institutional guidelines and with the approval of RIKEN committee. All patients have provided informed consent according to the institutional regulations.

## Author Contributions

ND, TS, and MA performed experiments; TT synthesized RK; ND, TC, KH, HS, AK, and MT analyzed and interpreted the data; ND, TC, HO, KH, and MT designed the experiments; ND and MT wrote the manuscript; MT supervised the work.

## Conflict of Interest Statement

The authors declare that the research was conducted in the absence of any commercial or financial relationships that could be construed as a potential conflict of interest. The reviewer GC and handling editor declared their shared affiliation.
