# Supplementary material for: Natural Killer T Cell-Targeted Immunotherapy Mediating Long-term Memory Responses and Strong Antitumor Activity
Source: Front Immunol. 2017 Sep 25;8:1206. doi: 10.3389/fimmu.2017.01206 (PMC5622408; doi:10.3389/fimmu.2017.01206)
Supplement: Supplementary file 1 [file data_sheet_1.pdf]

frontiers

# *Supplementary Material*

## NKT cell-targeted cancer vaccine mediating long-term memory responses and strong antitumor activity

**Nyambayar Dashtsoodol, Tomokuni Shigeura, Takuya Tashiro, Minako Aihara, Toshihiro Chikanishi, Hiromi Okada, Keigo Hanada, Masaru Taniguchi***

*** Correspondence:** Dr. Masaru Taniguchi: masaru.taniguchi@riken.jp

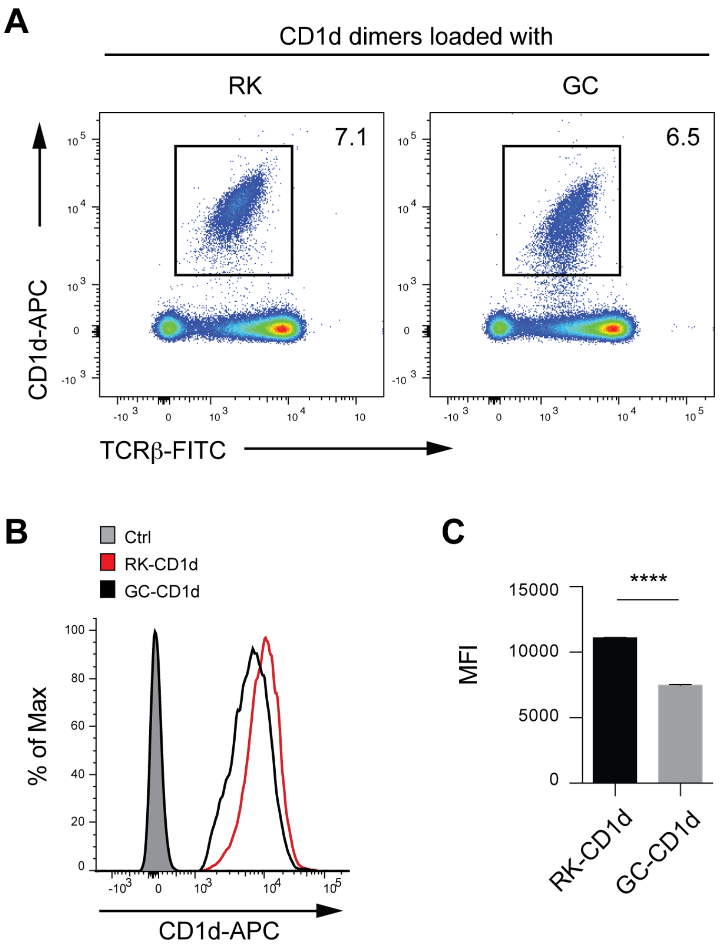


**Supplementary Figure 1. Staining of mouse NKT cells with RK-loaded CD1d dimers. (A)** Staining of WT B6 thymocytes with RK- or GC-loaded CD1d dimers. Numbers indicate percentage of CD1d dimer$^+$ TCRβ$^+$ cells within 7-AAD$^-$CD8$^-$ thymocytes. **(B)** Histogram overlay of RK-loaded (shown in red) with GC-loaded (shown in black) CD1 dimer$^+$ cells. Unloaded CD1d dimers were used as control staining (shown in gray). **(C)** The MFI levels of CD1d dimers loaded with RK or GC. Data are means ± SEM from three mice per group. All experiments were repeated three times with similar results. ****P<0.0001 with unpaired *t* test.
